# The Effect of Donor and Nonfullerene Acceptor Inhomogeneous Distribution within the Photoactive Layer on the Performance of Polymer Solar Cells with Different Device Structures

**DOI:** 10.3390/polym9110571

**Published:** 2017-11-03

**Authors:** Yaping Wang, Zhenzhen Shi, Hao Liu, Fuzhi Wang, Yiming Bai, Xingming Bian, Bing Zhang, Tasawar Hayat, Ahmed Alsaedi, Zhan’ao Tan

**Affiliations:** 1 State Key Laboratory of Alternate Electrical Power System with Renewable Energy Sources, North China Electric Power University, Beijing 102206, China; wyp614725@163.com (Y.W.); shizz123@126.com (Z.S.); dylht00@126.com (H.L.); wfz501@ncepu.edu.cn (F.W.); ymbai@ncepu.edu.cn (Y.B.); bianxingming@ncepu.edu.cn (X.B.); mpezb310@gmail.com (B.Z.); 2 Department of Mathematics, Quiad-I-Azam University, Islamabad 44000, Pakistan; tahaksag@yahoo.com; 3 NAAM Research Group, Faculty of Science, King Abdulaziz University, Jeddah 21589, Saudi Arabia; aalsaedi@hotmail.com

**Keywords:** organic solar cells, inhomogeneous distribution, non-fullerene photoactive layer, nickel oxide, anode buffer layer

## Abstract

Due to the inhomogeneous distribution of donor and acceptor materials within the photoactive layer of bulk heterojunction organic solar cells (OSCs), proper selection of a conventional or an inverted device structure is crucial for effective exciton dissociation and charge transportation. Herein, we investigate the donor and acceptor distribution within the non-fullerene photoactive layer based on PBDTTT-ET:IEICO by time-of-flight secondary-ion mass spectroscopy (TOF-SIMS) and scanning Kelvin probe microscopy (SKPM), indicating that more IEICO enriches on the surface of the photoactive layer while PBDTTT-ET distributes homogeneously within the photoactive layer. To further understand the effect of the inhomogeneous component distribution on the photovoltaic performance, both conventional and inverted OSCs were fabricated. As a result, the conventional device shows a power conversion efficiency (PCE) of 8.83% which is 41% higher than that of inverted one (6.26%). Eventually, we employed nickel oxide (NiO*_x_*) instead of PEDOT:PSS as anode buffer layer to further enhance the stability and PCE of OSCs with conventional structure, and a promising PCE of 9.12% is achieved.

## 1. Introduction

Bulk-heterojunction organic solar cells (BHJ-OSCs) attract increasing attention due to their characteristics of light-weight, low-cost, flexibility, rapid energy payback time and high-throughput roll-to-roll manufacturing [1,2,3]. Great efforts have been made in the development of new electron donors [4] and acceptors [5], the optimization of morphology [3], clean synthetic methods for the semiconductors production [6] and the engineering of device structure [7], resulting rapid promotion of the power conversion efficiency (PCE) of the OSCs. The PCE of fullerene-based OSCs is over 11%. However, further improvement in fullerene-based OSCs is facing great challenge due to their large energy losses. Recently, non-fullerene small molecules and all-polymer (using a *p*-type donor polymer and an *n*-type acceptor polymer) solar cells have emerged. Compared with fullerene derivatives, non-fullerene *n*-type small molecule and polymer acceptors can be flexibly designed to match the donor polymer in molecular weight, absorption spectra and energy level, allowing a better device performance [8,9,10,11]. Up to date, the PCE of non-fullerene acceptor based devices has exceeded 13% [5].

The photoactive layers in OSCs are commonly fabricated by casting the blend solution of electron donor and acceptor, which are dissolved in one or mixed solvents. Ideally, the donor and acceptor will homogeneously distribute in both lateral and vertical direction through the active layer and they will not gather around one side to transport certain electrons or holes. However, in reality, the distribution of the donor and acceptor within the photoactive layer is inhomogeneous due to the different surface energy and compatibility. The vertical concentration distribution was observed in fullerene based blend systems caused by the surface energy difference [12,13,14,15,16,17]. Take the P3HT:PC_60_BM OSC as an example, it is found that P3HT tends to enrich in the upper section of the photoactive layer due to the relatively lower surface energy (γ) of 16.9 mN/m, and a small amount of PCBM phase (γ = 45 mN/m) accumulates at the PEDOT:PSS interface [18]. This inhomogeneous distribution can also be found in non-fullerene and all-polymer solar cells such as PBDTTT-C-T:PDI and P3HT:F8TBT blends [19,20], where donors gather at the bottom and electrons enrich on the surface. Obviously, this kind of concentration distribution goes against the carrier transportation for conventional OSCs. While for inverted structure, the kind of component aggregation can offer continuous and unhindered pathways for easily carrier transportation to the corresponding electrode, thus enhancing device performance. Therefore, it is very important to understand the distribution of the donor and acceptor materials within the photoactive layer before choosing the conventional or inverted structure of OSCs. Recently, non-fullerene acceptor based OSCs have attracted increasing attention due to their adjustable energy level, facile synthesis, good solubility and wider absorption in the visible region. Thus, the investigation of concentration distribution in non-fullerene blends is crucial for further performance enhancement.

Herein, we investigate the vertical concentration distribution within the photoactive blend layer based on the non-fullerene acceptor IEICO and polymer donor PBDTTT-ET by time-of-flight secondary-ion mass spectroscopy (TOF-SIMS) and scanning Kelvin probe microscopy (SKPM). We find that the electron acceptor IEICO has lower concentration near the surface while the donor PBDTTT-ET has higher concentration near the surface. This inhomogeneous distribution is harmful to exciton dissociation and carrier transportation for inverted OSCs, but it is helpful for conventional structure, since the component aggregation can facilitate carrier transport to the corresponding electrode and enhance device performance. To confirm the vertical concentration distribution within the non-fullerene based photoactive layer, both conventional and inverted devices are designed and fabricated. For conventional OSCs, PEDOT:PSS and the low-work-function metal Ca are commonly used as anode and cathode interfacial materials, respectively. Since the acidic PEDOT:PSS could etch ITO and the low-work-function metals are sensitive to oxygen and water, resulting device degradation. It demonstrates that appropriate thickness of NiO*_x_* can not only suppress the interfacial energy losses between photoactive layer and ITO anode, but also can improve the stability of OSCs [21]. Thus, to further improve the device performance, air stable solution-processed nickel oxide (NiO*_x_*) and poly[(9,9-bis(3-((*N*,*N*-dimethyl)-*N*-ethylammonium)-propyl)-2,7-fluorene)-alt-2,7-fluorene)-alt-2,7-(9,9-dioctylfluorene)]dibromide (PFN-Br) are employed as anode and cathode interfacial materials, and a promising PCE of 9.12% is achieved for conventional device with high stability.

## 2. Experimental Section

### 2.1. Materials

Nickel acetate tetrahydrate, Titanium (diisopropoxide) bis (2,4-pentanedionate) (TIPD, 75% in isopropanol solution) and molybdenum trioxide (MoO_3_) were purchased from Alfa Aesar (Shanghai, China) and used directly without further purification. 1,8-diiodooctane (DIO) was purchased from Tokyo Chemical Industry (TCI). PBDTTT-ET, IEICO and PFN-Br were purchased from Solarmer Materials Inc. (Beijing, China).

### 2.2. Device Fabrication

The inverted OSCs were fabricated with the structure of ITO/TIPD (or TiO_2_)/PBDTTT-ET:IEICO/MoO_3_/Ag. ITO coated glass patterned with 2 mm width strips was ultrasonic cleaned twice by detergent, water, deionized water, acetone and isopropanol successively. TIPD was diluted by isopropanol for 20 times before spin-coated at a speed of 4000 rpm on the cleaned ITO glass and then baked at 150 °C for 10 min. Compact TiO_2_ layer was deposited by spray a 0.15 M titanium diisopropoxide bis(acetylacetonate) precursor solution using oxygen as carrier gas and then pyrolyzed at 450 °C for 30 min. Then the substrate was transferred into the nitrogen-filled glove-box for the following procedures. The photoactive layer was prepared by spin-coating PBDTTT-ET:IEICO chloroform solution (1:1.25 *w*/*w*, polymer concentration of 10 mg/mL) with 2% volume ratio of DIO additive on the ITO/TIPD (or TiO_2_) substrate. The thickness of the PBDTTT-ET:IEICO film is around 100 nm. Then the samples were transferred into a vacuum chamber, and a 10 nm of MoO_3_ layer and a 100 nm of Ag were thermally deposited on the photoactive layer under a base pressure of 5 × 10^−5^ Pa. The area of the device is around 4 mm^2^ defined by perpendicular cross area of ITO and cathode electrode.

For conventional OSCs, the device structure is ITO/NiO*_x_* (or PEDOT:PSS)/PBDTTT-ET:IEICO/PFN-Br/Al. The pre-cleaned ITO substrate was treated in an ultraviolet-ozone chamber (Ultraviolet Ozone Cleaner, Jelight Company, USA) for 15 min. The NiO*_x_* film was fabricated by spin-coating a nickel acetate tetrahydrate [Ni(CH_3_COO)_2_·4H_2_O] aqueous solution with different concentrations on the pretreated ITO substrate, followed by annealing in air at 150 °C for 10 min and UVO treatment for 4 min. PEDOT:PSS aqueous solution was filtered through a 0.45 μm filter and spin-coated at 4000 rpm for 30 s (30 nm) on the ITO electrode. Subsequently, the PEDOT:PSS film was baked at 150 °C for 15 min in air. The photoactive layer was prepared with the same experiment condition as that in the inverted device. Then, a cathode buffer layer of PFN-Br (1 mg/mL in methyl alcohol) was spin-coated at 4000 rpm for 30 s on the photoactive layer. Finally, a 100 nm of Al was thermally deposited in vacuum under at pressure of 5 × 10^−5^ Pa.

### 2.3. Device Characterization

The current density-voltage (*J–V*) measurement of the devices was conducted on a computer-controlled Keithley 2400 Source Measure Unit. *J–V* tests were done in a glove-box under simulated AM 1.5G irradiation (100 mW/cm^2^) using a xenon-lamp-based solar simulator (AAA grade, SAN-EI ELECTRIC Co., Ltd., Osaka, Japan). The external quantum efficiency (EQE) was measured using a Systems model QE-DLI lock-in amplifier coupled with a QE-M110 monochromator and 75 W xenon lamp (Enli Technology Co., Ltd., Kaohsiung, Taiwan). The light intensity at each wavelength was calibrated with a standard single-crystal Si photovoltaic cell. The EQE tests were performed under ambient condition.

### 2.4. Instrumentation

The surface morphologies of the PBDTTT-ET:IEICO photoactive layer and ITO substrates with different buffer layers were analyzed using an Agilent 5500 atomic force microscope (AFM) (Agilent, Santa Clara, CA, USA) operated in the tapping mode under ambient condition, the scan area was 5 μm by 5 μm. Scanning Kelvin probe microscopy (KPFM) measurements were also carried out on the same AFM using the standard SKPM mode. The secondary ion mass spectrum was obtained using a time-of-flight secondary ion mass spectrometer TOF-SIMS 5 from ION-TOF GmbH (Münster, Germany). An Ar^+^ sputter beam operating at a 5 keV beam voltage with a 45° incident angle was used. A depth profiling experiment was performed using an analysis beam of Bi^3+^ at a 30 keV beam voltage with a scanned area of 100 μm by 100 μm inside an etching area of 300 μm by 300 μm. The absorption and reflectance spectra of NiO*_x_* and PEDOT:PSS modified ITO substrates were measured from 300 to 900 nm by Shimadzu UV-2450 Spectrophotometer (Shimadzu, Kyoto, Japan). The slit width is 5.00 nm and scan speed is 300 nm/min. The reflectivity was measured using a Hitachi UH4150 UV–Vis spectrometer (Hitachi, Tokyo, Japan) with an integrating sphere (rectangular integration method). The slit width and scan speed are also 5.00 nm and 300 nm/min.

## 3. Results and Discussion

**Figure 1:**
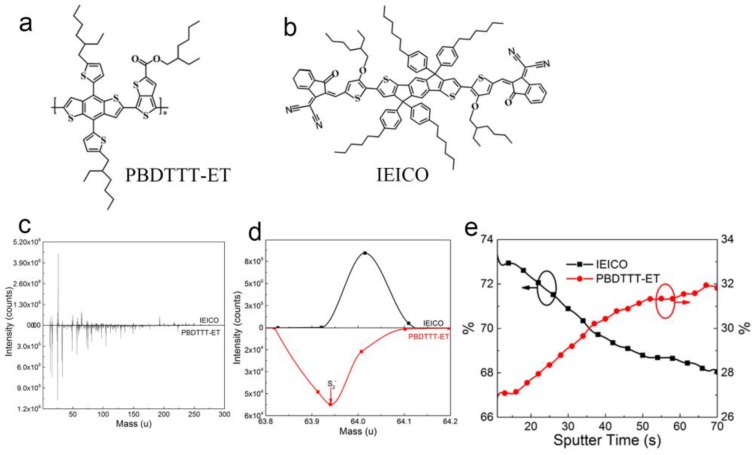
Molecular structures of (**a**) PBDTTT-ET and (**b**) IEICO; (**c**) distribution maps of elements within IEICO and PBDTTT-ET; (**d**) local enlarged drawing of distribution maps in (**c**); (**e**) depth profiles of IEICO and PBDTTT-ET in the blend film.

The molecular structures of PBDTTT-ET and IEICO are depicted in Figure 1a,b, respectively. To clarify the donor and acceptor distribution within the photoactive layer, time-of-flight secondary-ion mass spectroscopy (TOF-SIMS) measurements were conducted. TOF-SIMS is a surface analytical technique that focuses a pulsed beam of primary ions onto a sample surface, producing secondary ions in a sputtering process. TOF-SIMS can be used for depth profiling and complements dynamic SIMS. The advantages for profiling are its small areas capabilities and also its ability to do survey depth profiles without selecting specific elements of interest. Since it directly detects intrinsic sputtered secondary ions, TOF-SIMS is more accurate in compared with the traditionally used reflectivity measurements such as X-ray reflectivity (XRR), and neutron reflectivity (NR) [22]. Comparing the molecular structure of PBDTTT-ET with IEICO, nitrogen (N) is the characteristic element of acceptor IEICO, but there is no specific element for donor PBDTTT-ET. So we firstly investigated the characteristic species of PBDTTT-ET and IEICO by TOF-SIMS, and the distribution maps of elements within IEICO and PBDTTT-ET are shown in Figure 1c,d is the local enlarged drawing of Figure 1c. The molecular weight of highest percentage of sulfur isotopes is 31.97, S_2_^−^ is 63.94, as shown in Figure 1d. Hence, S_2_^−^ and ^13^CN^−^ are the specific species for PBDTTT-ET and IEICO, respectively. Then, we investigated the vertical distribution of S_2_^−^ and ^13^CN^−^ to explore the aggregation of PBDTTT-ET and IEICO through the photoactive layer by TOF-SIMS. To more directly illustrate the donor and acceptor distribution within the photoactive layer, the distribution is expressed as a percentage, as shown in Figure 1e. It can be seen that the IEICO decreases from 73% to 28%, while the PBDTTT-ET increases from 67% to 32% with the increasing sputter time. To intuitively observe the change of IEICO distribution in the photoactive layer surface, the individual distribution of ^13^CN^−^ (IEICO) on the surface and at the bottom of the active layer is displayed in Figure S1a,b (see Supplementary Materials), respectively, in which the white dots represent ^13^CN^−^. The peak area of ^13^CN^−^ on the surface and at the bottom are 8382 and 4503, respectively, which means that the concentration of IEICO on the surface is more than two times higher than that of at the bottom within the photoactive layer. Therefore, from the viewpoint of charge transportation, the PBDTTT-ET:IEICO blend is more suitable for conventional device structure where more IEICO enriches on the top surface, which could benefit electron transport and collection, at the same time, low concentration distribution of IEICO at the bottom could enhance hole transport and collection.

To further confirm the inhomogeneous distribution of donor and acceptor within the photoactive layer could greatly impact on the device performance with different structure, inverted (Figure S2a) and conventional (Figure S2b) OSCs are designed and fabricated. In order to avoid the influence of cathode buffer layer on the performance of inverted OSCs, two common interfacial materials, TiO_2_ and TIPD, were chosen to incorporate inverted OSCs, whose energy level match well with the PBDTTT-ET:IEICO photoactive layer. For conventional OSCs, PEDOT:PSS and the low-work-function metal Ca are commonly used as anode and cathode interfacial materials. However, the acidic PEDOT:PSS would etch ITO and cause morphology uniformity and chemistry instability [23,24,25]. The low-work-function metal Ca is sensitive to oxygen and water, leading to device degradation [26]. Hence, we employed solution-processed NiO*_x_* [27] and PFN-Br [28] as anode and cathode interfacial materials, respectively.

**Figure 2:**
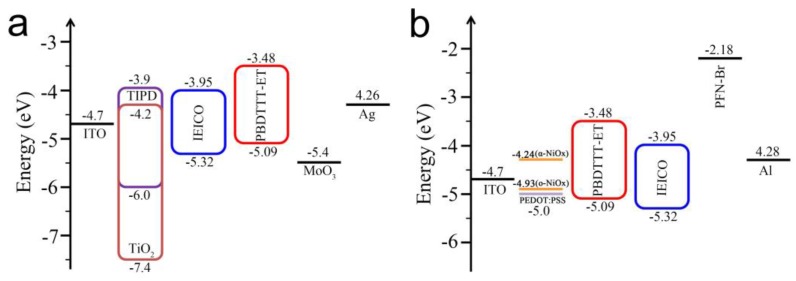
Energy level diagrams of (**a**) inverted and (**b**) conventional OSCs.

Figure 2a,b gives the energy diagrams of inverted and conventional devices. The electronic energy levels of TiO_2_ [29], TIPD [30], PBDTTT-ET:IEICO [31] and the work function of NiO*_x_* [27], PEDOT:PSS [32], MoO_3_ [7] and PFN-Br [4] are taken from literatures. As shown in Figure 2a, the lowest unoccupied molecular orbital (LUMO) of TiO_2_ is −4.2 eV which aligns between IEICO (3.95 eV) and ITO (4.7 eV), promoting the electrons transport from IEICO to ITO cathode. The LUMO of TIPD (−3.9 eV) is almost the same as IEICO, thus the electrons can easily transport to the ITO through TIPD. Furthermore, both TiO_2_ and TIPD have a deep highest occupied molecular orbital (HOMO), −7.4 and −4.2 eV respectively, which can block the holes transportation and reduce carrier recombination. MoO_3_ can form an ohmic contact with the PBDTTT-ET donor material due to its high work function of 5.4 eV [33]. Therefore, from the viewpoint of energy level, this kind of inverted OSC is workable. For conventional OSCs as shown in Figure 2b, the NiO*_x_* film was prepared by spin-coating aqueous solution of nickel acetate tetrahydrate and then annealed at a low temperature of 150 °C for 10 min followed by UVO treated for 4 min, it is denoted as α-NiO*_x_* and o-NiO*_x_* before and after UVO treatment [27]. The o-NiO*_x_* film obtained an increased work function of 4.93 eV, much higher than that of α-NiO*_x_* (4.24 eV), resulting in better alignment with the HOMO of PBDTTT-ET and expected to obtain a higher open-circuit voltage (*V_oc_*). With 4.93 eV work function and 2.1 eV electron affinity, NiO*_x_* can not only effectively transport holes, but also block electrons in conventional devices [27,34,35]. Meanwhile, PFN-Br can introduce effective interface dipoles to reduce voltage losses [36,37].

**Figure 3:**
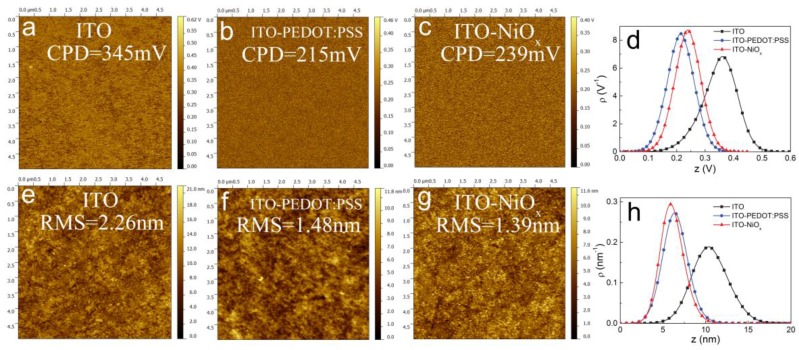
Contact potentials and AFM topography images of (**a**,**e**) ITO, (**b**,**f**) ITO/PEDOT:PSS and (**c**,**g**) ITO/NiO*_x_* (1.5 mg/mL); The normalization of the height density distributions of (**d**) contact potentials and (**h**) AFM topography images.

The optical, electronic and surface properties of the NiO*_x_* thin films obtained from our approach are also investigated. Figure 3 displays the surface potential and topography morphologies of NiO*_x_* films measured by scanning Kelvin probe microscopy (SKPM) and atomic force microscopy (AFM). During the SKPM measurement, the sample and tip is connected by a 0.5 mm platinum filament, and a direct current (DC) voltage is applied to the tip. The DC voltage will automatically adjust until the electrostatic force between the surface of the sample and the tip is nullified. In addition, the DC can be calculated by the following formula [38,39].

(1) $${\mathrm{CPD}\;=\;V}_{\mathrm{dc}}\;=\;\frac{\emptyset_{\mathrm{tip}}-\emptyset_{\mathrm{sample}}}{q}$$

where CPD is the abbreviation of contact potential difference (CPD), q is the electron charge, ∅_tip_ is the tip work function and ∅_sample_ is the work function of measured sample. We can infer from the above formula that the work function is inversely proportional to CPD. The CPD values calculated from Formula (1) for ITO, ITO/PEDOT:PSS and ITO/NiO*_x_* substrates are 345, 215 and 239 mV, respectively, as shown in Figure 3a–c, indicating that the work function of ITO is greatly enhanced with PEDOT:PSS or NiO*_x_* modification, which is more favorable to the formation of ohmic contact between the anode and photoactive layer, thus leading to a higher *V_oc_*. Furthermore, we utilized a height distribution function, which is computed as normalized histograms of the height values, to illustrate the surface potential differences between ITO, ITO/PEDOT:PSS and ITO/NiO*_x_* directly, as shown in Figure 3d. Where ρ(p) is the normalization of the density, and p is the corresponding quantity height.

(2) $$\int_{-\infty }^{\infty }\rho (p)\mathrm{dp}\;=\;1$$

Observing from the topography morphologies as shown in Figure 3e–h, we can find that the ITO substrate becomes smoother after modified with NiO*_x_*, and the root mean square roughness (RMS) of ITO decreases from 2.26 to 1.39 nm, which is even smoother than that of PEDOT:PSS modified surface (1.48 nm). The results indicate that a smoother interfacial contact can be obtained between the photoactive layer and anode, which is beneficial to the exciton separation and charge transportation, thus increasing the short-circuit current density (*J_sc_*) [40]. We also calculated the height distribution of AFM topography images with the same statistical function as the Formula (2), and the result is shown in Figure 3h. Obviously, the NiO*_x_* modified surface demonstrates the lowest surface roughness.

**Figure 4:**
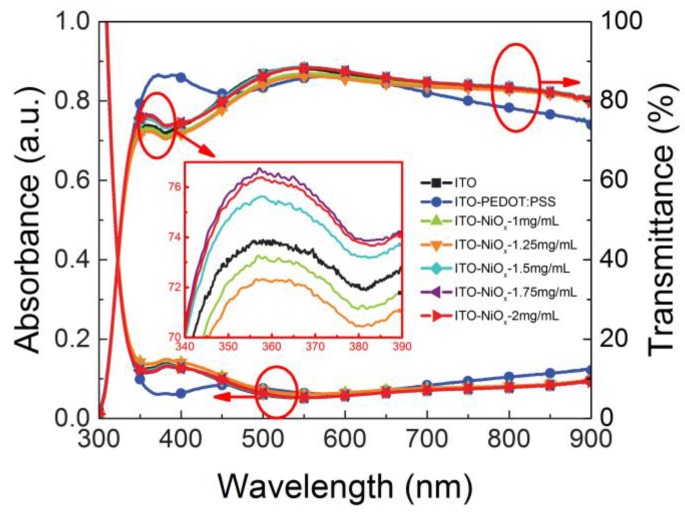
Optical absorption and transmittance spectra of ITO, ITO/PEDOT:PSS and ITO/NiO*_x_* (with different concentration) substrates, the inset is the local enlarged image of 340–390 nm.

As anode interfacial material, apart from the surface properties, optical characteristics of NiO*_x_* are equally important. Thus, we carried out the absorption and transmission tests and the results are shown in Figure 4, the inset is the local enlarged image (340–390 nm) of bare ITO and different concentration NiO*_x_* coated ITO. In comparison with bare ITO substrate without any modification layer, the absorption of NiO*_x_* coated ITO substrate is almost unchanged in the whole visible wavelength range. The corresponding transmittance is basically unchanged in addition to a slight increase in the short wavelength, and still maintains a high degree of transparency in the visible and near-infrared region, with an average transmittance of about 85%. In the short wavelength of 350–450 nm, the PEDOT:PSS coated ITO is more transmissive than that of bare ITO. Such anti-reflection effect should be attributed to the optical interference between the organic layer (PEDOT-PSS) and inorganic (ITO) layer due to their large refractive index (*n*) difference [41]. Compared with PEDOT:PSS film, the NiO*_x_* film even has lower absorption and higher transmittance in 650–900 nm wavelength range due to the different optical constants of organic and inorganic materials. Furthermore, as depicted in the inset figure, fine-tune the concentration of NiO*_x_* from 1 to 2 mg/mL has little influence on the absorption and transmittance. This satisfactory property lays a good optical foundation for the application of NiO*_x_* as anode interfacial material in OSCs.

**Figure 5:**
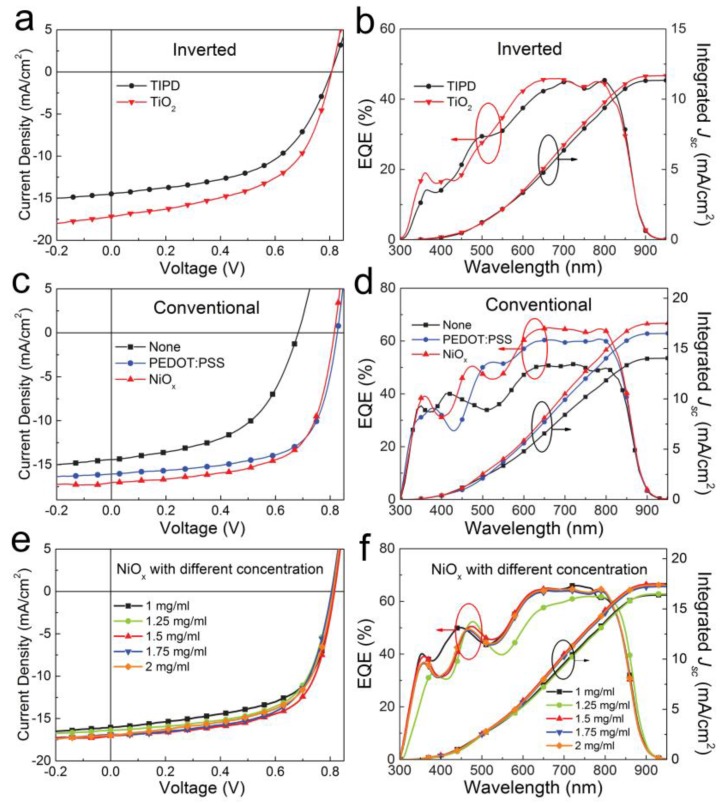
(**a**) Current density−voltage (*J–V*) and (**b**) EQE curves of inverted OSCs with TIPD and TiO_2_ as cathode buffer layers; (**c**) *J–V* and (**d**) EQE curves of conventional OSCs without anode buffer layer, with PEDOT:PSS and with NiO*_x_* as anode buffer layers; (**e**) *J–V* and (**f**) EQE curves of conventional OSCs with different thickness of NiO*_x_* film as the anode buffer layer.

The current density-voltage (*J–V*) curves and external quantum efficiency (EQE) curves of OSCs with different device structures are shown in Figure 5. The key performance parameters of *V_oc_*, *J_sc_*, filling factor (FF) and PCE are summarized in Table 1. As displayed in Figure 5a, under illumination, the inverted OSC with TIPD as the buffer layer exhibits an average PCE of 6.26%, with a *V_oc_* of 0.81 V, a *J_sc_* of 14.48 mA/cm^2^ and an FF of 53.6%, and the TiO_2_-based device shows a higher *J_sc_* of 16.01 mA/cm^2^, a same *V_oc_* of 0.81 V, a similar FF of 52.8% and an overall PCE of 6.86%. As presented in Figure 5b, the integrated *J_sc_* calculated from EQE curves are 11.31 and 11.74 mA/cm^2^ respectively for TIPD and TiO_2_-based devices, and there is some mismatch between the *J_sc_* obtained from *J–V* curve and the integrated current calculated from EQE curve, which is probably due to the inside defects of the photoactive layer [42,43,44]. To further understand the cause of the differences between the efficiencies of conventional and inverted devices, IQE is given by IQE = EQE/(1 − R), where EQE is the external quantum efficiency and R is the reflectivity, as shown in Figure S3. The IQE is inversely related to the amount of recombination that is occurring in the cell. As shown in Figure S3, the IQE of conventional OSC exhibits a high IQE about 80% from 500 to 850 nm and much higher than that of inverted one, which validates the efficient photon conversion properties of conventional OSC. Furthermore, the surface morphology of PBDTTT-ET:IEICO photoactive layer deposited on ITO/TIPD and ITO/PEDOT:PSS substrates are investigated by AFM measurements as shown in Figure S4. We can see that the height and phase morphologies of ITO/TIPD/PBDTTT-ET:IEICO and ITO/PEDOT:PSS/PBDTTT-ET:IEICO are remain similar, and the RMS is 6.05 and 6.02 nm, respectively, and the difference is negligible. As we can see from 3D views in Figure S4, the crystallization of conventional and inverted photoactive layer in the upper 50 nm shows no significant difference. These observations confirm that the performance differences between the conventional and inverted devices are not due to the morphology difference.

Figure 5c, d illustrates the *J–V* and EQE curves of conventional OSCs with different anode buffer layers. As depicted in Figure 5c, the control device without anode buffer layer shows a *J_sc_* of 14.89 mA/cm^2^, a *V_oc_* of 0.69 V and an FF of 52.9%, leading to an overall PCE of 5.43%. For device with PEDOT:PSS as anode buffer layer, the better performance can be achieved, with a *J_sc_* of 16.54 mA/cm^2^, a *V_oc_* of 0.82 V, an FF of 65.1%, and a PCE of 8.83%. As expected, the device with NiO*_x_* anode buffer layer shows the best PCE of 9.12%, with a *V_oc_* of 0.81 V, a *J_sc_* of 17.52 mA/cm^2^ and an FF of 64.3%. Compared with the PEDOT:PSS based device, the PCE improvement for NiO*_x_* based device is mainly ascribed to the *J_sc_* enhancement, and the *J_sc_* enhancement should be attributed to the optical spacer effect induced by NiO*_x_* film due to different optical constants of organic and inorganic materials [45,46,47]. The NiO*_x_* film can modulate the light distribution within the whole active layer, subsequently impact the *J_sc_*. Figure 5d gives the EQE curves of conventional OSCs with different buffer layers. The integrated *J_sc_* calculated from EQE curves are 14.04, 16.49 and 17.52 mA/cm^2^ for the devices without any anode buffer layer, with PEDOT:PSS and NiO*_x_* as anode buffer layer, respectively, which is in good agreement with the *J_sc_* obtained from *J–V* curves. The OSCs with anode buffer layers show much higher EQE than that of OSCs without anode buffer layer in almost the whole wavelength which is consistent with the higher *J_sc_*. Furthermore, the NiO*_x_* OSC obtains the highest integrated current of 17.52 mA/cm^2^, and the EQE enhancement is mainly in 300–500 nm and 575–900 nm spectral ranges.

Obviously, the device performances of conventional structure are much higher than that of inverted ones. This is an indirect evidence of the aforementioned vertical concentration distribution as shown in Figure S2, since more IEICO gathers on the top surface and low concentration of IEICO distributes at the bottom, which could benefit charges transport and collection, resulting higher performance of conventional devices.

Furthermore, we carefully tuned the thickness of NiO*_x_* film to further optimize the device performance. The *J–V* and EQE curves of OSCs with different thickness of NiO*_x_* layers are given in Figure 5e,f, and the key performance parameters are summarized in Table 2. With increasing concentration of NiO*_x_*, the *J_sc_* increases first and then remains at a certain level, the *V_oc_* is almost constant and the FF increases first and then decrease. Actually, tuning the thickness of NiO*_x_* layer is finding the tradeoff between the charge transport and surface coverage. Too thinner film cannot cover the whole ITO surface and too thicker film should increase the contact resistance. An overall optimal PCE of 9.12% was achieved at a concentration of 1.5 mg/mL, with a *V_oc_* of 0.81 V, a *J_sc_* of 17.52 mA/cm^2^ and an FF of 64.3%. As depicted in Figure 5f, the integrated *J_sc_* calculated from EQE curves is 16.38, 16.50, 17.50, 17.22 and 17.39 mA/cm^2^ respectively for devices with 1, 1.25, 1.5 1.75 and 2 mg/mL NiO*_x_*, which is consistent with the *J_sc_* value obtained from the *J–V* curves of the corresponding devices.

**Figure 6:**
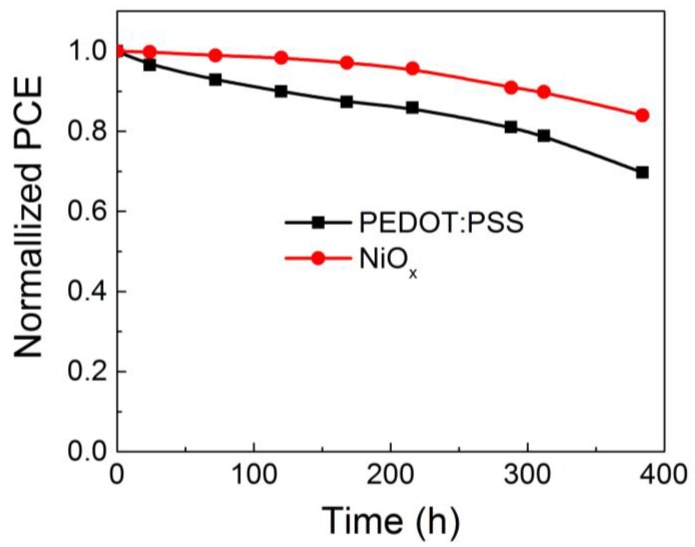
Long-term stability of the PCE for unencapsulated OSCs based on either PEDOT:PSS or NiO*_x_* anode buffer layers. The devices have been stored in N_2_ filled glove box.

Since we employed NiO*_x_* film to substitute the acidic and hydrophilic PEDOT:PSS, the OSCs should have a better stability as expected. Thus, long-term stability of unencapsulated OSCs fabricated with PEDOT:PSS and NiO*_x_* anode buffer layers were investigated by testing the device performance every 24 h in N_2_ filled glove box. Figure 6 shows the degradation behaviors of the normalized PCEs of both devices. It can be seen that OSCs with NiO*_x_* buffer layer demonstrated better stability. For the devices based on PEDOT:PSS buffer layer, the PCE drops to less than 70% of its initial value, while the OSCs based on NiO*_x_* buffer layer still remains 85% initial value after 16 days. The stability improvement can be attributed to the optimized interface between anode and photoactive layer. Therefore, NiO*_x_* was demonstrated a promising anode buffer layer for enhancing the environmental stability of solution-processed OSCs.

## 4. Conclusions

In conclusion, we have confirmed there is vertical concentration distribution within the nonfullerene based photoactive layer PBDTTT-ET:IEICO by TOF-SIMS measurements and device characterization. TOF-SIMS results indicate that the acceptor IEICO enriches on the top surface and the donor PBDTTT-ET uniformly distributes throughout the photoactive layer. This spontaneous vertical concentration distribution favors the charge transportation of device with conventional structure, and OSCs with conventional structure show significant enhancements in *J_sc_* and FF, leading to a 41% enhancement in PCE compared with the inverted ones. More stable and efficient devices have been achieved by introducing solution-processed NiO*_x_* as anode interfacial material in conventional OSCs. Compared with PEDOT:PSS based devices, NiO*_x_* based devices demonstrate increased *J_sc_* and longer lifetime, the *J_sc_* increases from 16.54 to 17.52 mA/cm^2^ due to the increasing charge extraction efficiency, and the PCE still remains 85% of its initial value after stored for 16 days, while the PEDOT:PSS based OSCs decays to below 70% of its initial value. Our findings indicate that, based on the inhomogeneous distribution of donor and acceptor materials within the photoactive layer, properly selection the conventional or inverted device structure is crucial for effective exciton dissociation and charge transportation. Furthermore, solution-processed NiO*_x_* is a promising alternative to PEDOT:PSS for fabrication of efficient and stable OSCs.

## Figures and Tables

**Table 1:** Device parameters of OSCs with different structure and different buffer layers.

Device Structure		*J_sc_* (mA/cm^2^)	*V_oc_* (V)	FF (%)	PCE (%) Average ^1^
Inverted	TIPD	14.48	0.81	53.6	6.26 (6.15)
	TiO_2_	16.01	0.81	52. 8	6.86 (6.73)
Conventional	None	14.89	0.69	52.9	5.43 (5.24)
	PEDOT:PSS	16.54	0.82	65.1	8.83 (8.77)
	NiO*_x_*	17.52	0.81	64.3	9.12 (9.06)

^1^ The average values were obtained from 20 devices.

**Table 2:** Device parameters of OSCs with different thickness of NiO*_x_* layer as the anode buffer layer.

NiO*_x_* Concentration (mg/mL)	*J_sc_* (mA/cm^2^)	*V_oc_* (V)	FF (%)	PCE (%) Average ^1^
1	16.38	0.81	59.7	7.92 (7.79)
1.25	16.50	0.80	62.1	8.20 (8.11)
1.5	17.52	0.81	64.3	9.12 (9.06)
1.75	17.37	0.80	62.4	8.68 (8.47)
2	17.46	0.80	60.5	8.46 (8.32)

^1^ The average values were obtained from 20 devices.

## Supplementary Materials

The following are available online at www.mdpi.com/2073-4360/9/11/571/s1, Figures S1–S4.
