# Supplementary material for: The Effect of Donor and Nonfullerene Acceptor Inhomogeneous Distribution within the Photoactive Layer on the Performance of Polymer Solar Cells with Different Device Structures
Source: Polymers (Basel). 2017 Nov 3;9(11):571. doi: 10.3390/polym9110571 (PMC6418818; doi:10.3390/polym9110571)
Supplement: Supplementary file 1 [file polymers-09-00571-s001.pdf]

Supplemrntary materials: The Effect of Donor and Nonfullerene Acceptor Inhomogeneous Distribution within the Photoactive Layer on the Performance of Polymer Solar Cells with Different Device Structures

Yaping Wang, Zhenzhen Shi, Hao Liu, Fuzhi Wang, Yiming Bai, Xingming Bian, Bing Zhang, Tasawar Hayat, Ahmed Alsaedi and Zhan'ao Tan

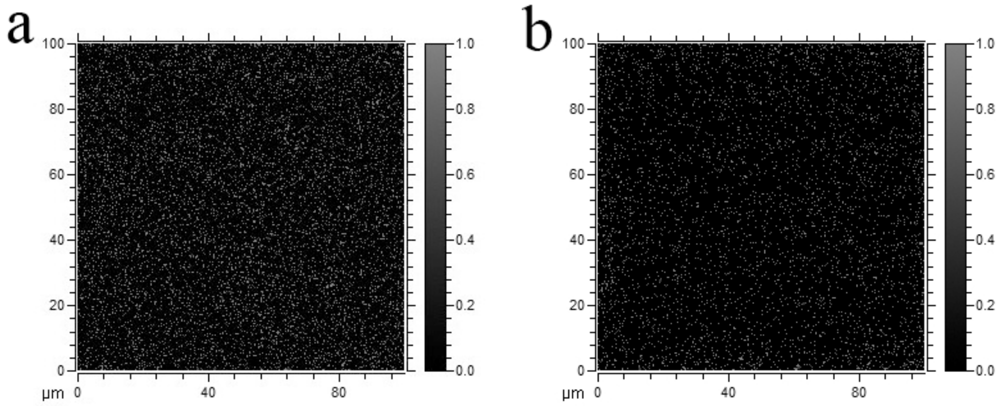

Figure S1. The individual distribution of $^{13}CN^-$ (IEICO) (a) on the surface and (b) at the bottom of the photoactive layer, in which the white dots represent $^{13}CN^-$.

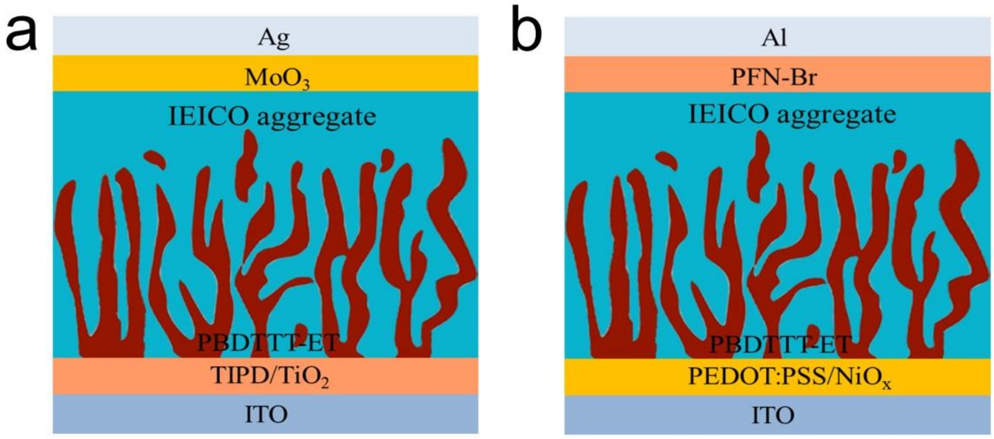

Figure S2. Schematic diagrams of (a) inverted and (b) conventional structure OSCs.

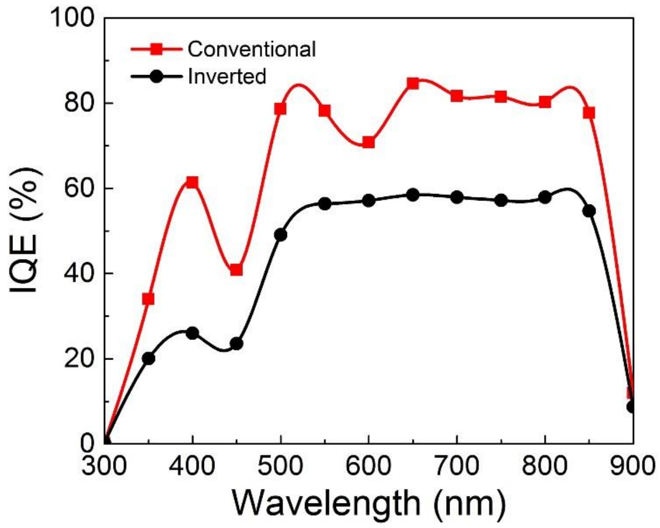

Figure S3. IQE curves of conventional and inverted OSCs.

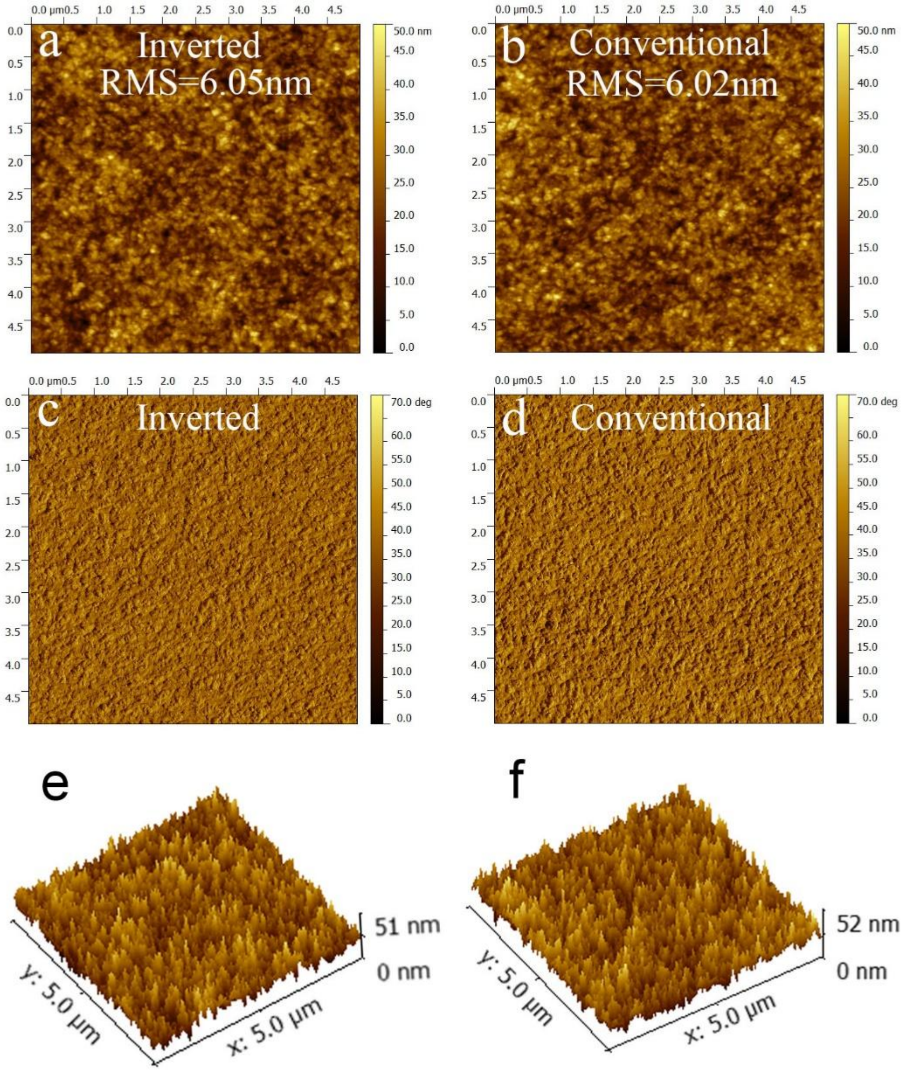

Figure S4. AFM height, phase and 3D view of topography images of PBDTTT-ET:IEICO deposited on (a, c, e) ITO/TIPD , (b, d, f) ITO/PEDOT:PSS substrates.
